# Complete Genome Sequences of Four Putatively Antibiotic-Producing Bacteria Isolated from Soil in Arkansas, USA

**DOI:** 10.1128/MRA.00745-21

**Published:** 2022-01-06

**Authors:** Ruth Plymale, Griffin Hopkins, Taylor Johnson, Taylor Savage, Danielle Schaal

**Affiliations:** a Department of Biology, Ouachita Baptist University, Arkadelphia, Arkansas, USA; University of Rochester School of Medicine and Dentistry

## Abstract

Soil bacteria can be a valuable source of antimicrobial compounds. Here, we report the complete genomes of four soil bacteria that were isolated by undergraduate microbiology students as part of a course-based research experience. These genomes were assembled using a hybrid approach combining paired-end Illumina reads with Oxford Nanopore Technologies MinION reads.

## ANNOUNCEMENT

Soil ecosystems have historically been good sources of antibiotic-producing bacteria (1) and, although genome mining has shown promise for antibiotic discovery (2), the culture and screening of soil bacteria may still be valuable (3). These four strains were selected for their antagonistic activity against two or more ESKAPE (Enterococcus faecium, Staphylococcus aureus, Klebsiella pneumoniae, Acinetobacter baumannii, Pseudomonas aeruginosa, and Enterobacter species) pathogens (4) as part of a course-based microbiology research experience (5). Soil samples were taken at the global positioning system (GPS) coordinates indicated in Table 1. Strain isolation was achieved by diluting soil samples in 1× phosphate-buffered saline (PBS), incubating each dilution aerobically on the isolation agar listed in Table 1 at 25°C for 5 days, and streaking individual colonies on tryptic soy agar (TSA) and growing them aerobically at 25°C for 2 days. Once these strains were selected for further study, each strain was grown on two TSA plates at 25°C for 2 days. One plate of each strain was shipped overnight to Genewiz (South Plainfield, NJ) for 16S rRNA sequencing of a colony from each plate, targeting the V1 through V9 regions; these sequences were analyzed for genus identification using NCBI BLAST with the 16S rRNA sequence database (6). The bacterial growth on the second plate for each strain was scraped into brain heart infusion broth with 25% glycerol and frozen at −80°C. Each strain was grown from frozen stock on TSA at 25°C for 2 days, and the plates were shipped overnight to the Microbial Genome Sequencing Center (MiGS) (Pittsburgh, PA), where a colony was removed from each plate for DNA extraction and sequencing. Genomic DNA was extracted using a combination of lysozyme and proteinase K enzymatic treatments before finishing with a Qiagen blood and tissue kit. The same genomic DNA isolation of each strain was used for both long- and short-read sequencing with Oxford Nanopore Technologies (ONT) and Illumina systems, respectively (7). Default parameters were used for all software. For ONT sequencing, libraries were prepared using kit SQK-LSK109 according to the manufacturer’s specifications (no DNA size selection/shearing), sequencing was performed with a MinION R9 flow cell, and base calling was performed using Guppy v4.2.2 (GPU mode) (8). An Illumina Nextera kit was used with modifications, as described by Baym et al. (9), to prepare Illumina libraries, which were sequenced on a NextSeq 550 platform; bcl2fastq v2.20.0.422 was used for demultiplexing, quality control, and trimming of the Illumina paired-end reads (2 × 150 bp) (10), Porechop v0.2.4 for quality trimming and removal of adapters for ONT sequencing (11), and Unicycler v0.4.8 for hybrid *de novo* assembly, circularization, and rotation to locate *dnaA* at nucleotide 1 (12). Each chromosome or plasmid was assembled into a single contig. Sequencing and assembly details are given in Table 1. The assembled genomes were annotated by the NCBI Prokaryotic Genome Annotation Pipeline (PGAP) v5.1 (13).

**TABLE 1:** Sequencing and assembly details for the isolated strains

Strain	GPS coordinates	Isolation agar	GenBank accession no.	Genome size (Mbp)	No. of contigs	SRA accession no. for ONT reads	No. of trimmed ONT reads	ONT read coverage (×)	*N*_50_ (Mbp)	SRA accession no. for Illumina reads	No. of Illumina read pairs	Illumina read coverage (×)	GC content (%)
Bacillus halotolerans strain MBH1	34.124260, −93.050649	TSA	CP070976	4.23	1	SRX11445848	192,595	338	4.23	SRX11445847	3,145,590	216	43.59
*Burkholderia* sp. strain LAS2	34.124167, −93.061667	TSA	CP071052, CP071053, CP071054, CP071055, CP071056	7.68	5	SRX11443407	97,395	190	4.46	SRX11443406	2,594,142	99	66.89
Pseudomonas entomophila strain Small	34.144506, −93.057681	Potato dextrose agar	CP070982	5.96	1	SRX11443507	194,084	220	5.96	SRX11443506	2,867,625	140	63.94
Pseudomonas rhodesiae strain AAMF24	39.3102, −93.1519	TSA	CP070980	5.772	1	SRX11444531	159,922	216	5.77	SRX11444530	2,987,467	151	60.49

### Data availability.

All sequences were deposited in the NCBI GenBank database under BioProject accession number PRJNA673262. The assembled genomes and ONT and Illumina reads are available under the accession numbers listed in Table 1.
